# Comparative analysis of marginal bone loss around implant restored with platform switched versus platform matched abutments

**DOI:** 10.6026/973206300221029

**Published:** 2026-02-28

**Authors:** P Shashank, Santhosh Kumar Kuna, Sravanthi Jagati, E Sravanthi, Zakirulla Meer, Ruparani Bodduru, Naseemoon Shaik

**Affiliations:** 1 Department of Head and Neck, Padhar Hospital, Madhya Pradesh, India; 2 Department of Oral and Maxillofacial Surgery, Consultant at King Faisal Hospital, Rwanda, Centre Hospitalier Universitaire de Kigali, Rwanda; 3 Department of Pedodontics and Preventive Dentistry, MNR Dental College and Hospital, Sangareddy, Telangana, India; 4 Department of Prosthodontics, Army College of Dental Sciences, Hyderabad, Telangana, India; 5 Department of Pediatric Dentistry and Orthodontic Sciences, College of Dentistry, King Khalid University, Abha, Saudi Arabia; 6 Department of Periodontics, MNR dental College and Hospital, Sangareddy, Telangana, India

**Keywords:** Abutment design, dental implants, marginal bone loss, platform matching, platform switching

## Abstract

Marginal bone loss around dental implants remains a persistent clinical challenge affecting long-term implant success. This study compared crestal bone changes in 100 patients restored with platform-switched versus platform-matched abutments over a 12-month period. Standardized radiographs revealed significantly lower marginal bone loss in the platform-switched group at every follow-up interval. Clinical parameters remained stable in both groups, confirming that inflammation did not influence bone-level differences. Thus data shows clear superiority of platform switching in preserving peri-implant bone.

## Background:

Marginal bone loss around dental implants remains one of the most clinically relevant parameters determining implant success, long- term stability and peri-implant health. Although osseointegrated implants demonstrate high survival rates, the early crestal bone remodeling that occurs after implant placement and loading can influence long-term prognosis and esthetic outcomes, especially in the anterior region [1]. Multiple biological and mechanical factors including surgical trauma, microgap location, implant-abutment connection design, biomechanical loading and peri-implant soft tissue characteristics contribute to changes in marginal bone height over time [2]. One important concept introduced to address crestal bone preservation is platform switching, a design in which the abutment diameter is intentionally smaller than the diameter of the implant platform. This inward horizontal offset displaces the implant-abutment microgap away from the crestal bone, theoretically reducing inflammatory cell infiltration and minimizing bone remodelling [3]. Biomechanically, the configuration redistributes occlusal forces toward the central axis of the implant, decreasing stress concentration at the crestal bone. Biological theories also suggest that platform switching allows for a more stable connective tissue attachment by providing space for biologic width establishment without encroaching on the bone crest [4]. In contrast, platform matching implants use abutments with the same diameter as the implant platform, a traditional design that places the microgap at the outer edge of the implant-bone interface. This configuration may expose the crestal bone to greater inflammatory and mechanical challenges, potentially leading to comparatively higher marginal bone loss, especially after functional loading [5]. Although platform-matched abutments have been used reliably for decades, concerns related to crestal bone changes have encouraged clinicians and researchers to explore alternative implant-abutment interface designs [6]. Numerous clinical and radiographic studies have demonstrated that platform switching can reduce bone resorption, but the magnitude and consistency of its benefits vary across implant systems, follow-up periods and clinical protocols. Some investigations report significantly lower bone loss with platform switching, while others show minimal differences when compared with platform matching [7]. These discrepancies may arise from variability in implant design, connection type, peri-implant tissue thickness, surgical approach (immediate vs. delayed placement) and patient-related factors such as oral hygiene, smoking and systemic conditions. Additionally, radiographic evaluation techniques and timing of measurements differ across studies, influencing the comparability of results [8]. Given the increasing clinical emphasis on preserving peri-implant tissues and maintaining esthetic outcomes, determining the effectiveness of platform switching relative to platform matching becomes essential. Clear evidence supporting superior bone preservation can guide implant selection, abutment choice and treatment planning, particularly in areas where crestal bone stability is critical. Moreover, evaluating bone loss in a controlled, comparative manner contributes to standardizing protocols and optimizing long-term implant success rates [9]. Although platform switching has gained widespread acceptance, continued comparative research is required to clarify its true clinical advantage, quantify expected bone preservation and identify conditions under which it is most beneficial. With advancements in implant prosthetics and a growing focus on soft and hard-tissue stability, an evidence-based understanding of marginal bone behavior remains crucial for clinicians. Therefore, it is of interest to describe the clinical implications of platform switching in preserving marginal bone height, evaluate its comparative effectiveness and identify the conditions under which it provides the most significant benefit for long-term implant stability and esthetic outcomes.

## Methodology:

A prospective comparative clinical study was conducted on 100 partially edentulous patients requiring single-tooth implant-supported restorations to evaluate marginal bone loss around implants restored with platform-switched and platform-matched abutments. All participants, aged 20-65 years and having adequate bone volume for implant placement without extensive grafting, were selected based on predefined inclusion criteria, while patients with uncontrolled systemic diseases, active periodontal disease, heavy smoking habits, parafunctional habits, or a history of radiotherapy were excluded. The 100 eligible patients were divided into two equal groups of 50 each: Group I received platform-switched abutments and Group II received platform-matched abutments. Implant surgeries were performed by the same experienced surgeon under local anesthesia following standard aseptic protocols. A crestal incision and mucoperiosteal flap reflection were carried out and osteotomies were prepared using sequential drills under saline irrigation. Root-form implants of identical design and surface characteristics were placed at crestal bone level and primary stability was verified by insertion torque. Cover screws were placed, flaps were sutured and patients were recalled for suture removal after 7-10 days. After a healing period of 3 months in the mandible and 4-6 months in the maxilla, second-stage procedures were performed where needed and healing abutments were placed to allow soft tissue maturation. Impressions were then made and definitive abutments were selected according to group allocation: platform-switched abutments with a 0.3-0.5 mm horizontal mismatch for Group I and platform-matched abutments of equal diameter for Group II. All abutments were torqued to the manufacturer's recommended values and restored with standardized metal-ceramic or zirconia crowns. Standardized periapical radiographs were obtained using the paralleling technique at baseline (prosthetic loading), 3 months, 6 months and 12 months. Measurements of marginal bone levels were performed digitally by calibrating images using the known implant dimensions and the distance from the implant shoulder to the first bone-to-implant contact was recorded on mesial and distal sides. Clinical parameters including plaque index, bleeding on probing and probing depth were also recorded during each follow-up to ensure peri-implant tissue health and eliminate confounding inflammatory influences. The primary outcome was mean marginal bone loss at each interval, while secondary outcomes included peri-implant soft tissue parameters. All collected data were subjected to statistical analysis, with descriptive statistics calculated for each variable. Intergroup comparison of marginal bone loss at different intervals was performed using independent t-test or Mann-Whitney U test as appropriate, while intragroup changes over time were assessed using repeated-measures ANOVA or Friedman test and a p-value of <0.05 was considered statistically significant.

## Results:

A total of 100 patients completed the study, with 50 participants in the platform-switched group and 50 in the platform-matched group. All implants showed successful osseointegration without any biological or mechanical complications during the 12-month evaluation period. Baseline demographic characteristics, including age and gender distribution, were comparable between the two groups (Table 1). Clinical parameters such as plaque index, bleeding on probing and probing depth remained within clinically acceptable limits and showed no statistically significant differences between the groups throughout the follow-up period (Table 2), ensuring that marginal bone loss measurements were not influenced by peri-implant inflammation. Marginal bone levels at baseline, 3 months, 6 months and 12 months were recorded for both groups. The mean marginal bone loss was consistently lower in the platform-switched group compared with the platform-matched group at all follow-up intervals (Table 3). At 12 months, the platform-switched group demonstrated a mean bone loss of 0.52 ± 0.14 mm, whereas the platform-matched group showed 0.89 ± 0.21 mm and this difference was statistically significant (p < 0.001). Intragroup comparisons showed a progressive increase in bone loss from baseline to 12 months in both groups, although the magnitude of change was significantly lower in the platform-switched group (Table 4). Overall, the findings confirmed that platform switching effectively reduced crestal bone remodeling compared with platform matching over the 12-month period.

## Discussion:

The present study evaluated and compared marginal bone loss around implants restored with platform-switched and platform-matched abutments over a 12-month period. The findings demonstrated that implants restored with platform-switched abutments exhibited significantly less crestal bone loss than those with platform-matched abutments at all follow-up intervals. This supports the growing body of evidence suggesting that platform switching plays a beneficial role in preserving peri-implant crestal bone by relocating the implant-abutment microgap inward, thereby reducing the concentration of inflammatory infiltrate near the bone crest and improving biomechanical stress distribution. The reduced bone loss observed in the platform-switched group in the present study aligns with the findings of Lazzara and Porter (2006) [10], who first introduced the concept of platform switching and reported decreased bone remodeling around implants restored with smaller-diameter abutments. Their observations indicated that the inward horizontal offset created additional space for the establishment of a stable soft tissue seal, ultimately minimizing crestal bone resorption. Our results similarly showed that the mean bone loss at 12 months was significantly lower in the platform- switched group, supporting the original biological rationale proposed in that study. The current findings also correspond with the results of Guerra *et al.* (2014) [11], who observed that platform switching reduced bone loss by approximately 0.4 mm compared to platform matching. In our study, the difference between the two groups at 12 months was comparable, showing a mean difference of around 0.35 mm. Both studies highlighted that the beneficial effect persists regardless of implant site, provided that other clinical variables such as oral hygiene, soft tissue thickness and loading conditions are controlled. This consistency reinforces the clinical reliability of platform switching in various anatomical scenarios. Comparatively, Strietzel *et al.* (2015) [12] reported that platform switching offered significant advantages particularly during the early healing phase, with reduced bone remodeling occurring between prosthetic loading and three months.

Our findings also demonstrated that the greatest difference between the two groups emerged early, at the three-month interval, where platform switching reduced bone loss by nearly half compared with platform matching. This reinforces the hypothesis that early bone stability is a critical determinant of long-term success and that platform switching is most effective during this biologically sensitive phase. Additionally, Atieh *et al.* (2010) [13] conducted a systematic review concluding that platform switching reduced marginal bone loss by an average of 0.41 mm compared with platform-matched implants. The similarity between their pooled mean values and the results of this study confirms that the effect of platform switching is not limited to isolated clinical trials but is supported across a larger evidence base. Their review emphasized the need for standardized radiographic evaluation methods, which were incorporated in the present study through the use of the paralleling technique and calibrated digital measurements. Although the findings largely support the superiority of platform switching, it is important to note that factors such as soft tissue biotype, implant-abutment connection design, occlusal load and surgical protocol may influence marginal bone behavior. Some studies have reported minimal differences between platform switching and matching when peri-implant soft tissues are thick or when internal conical connections are used. In the present study, the use of identical implant systems, standardized surgical procedures and controlled loading conditions helped minimize these confounders, increasing the reliability of the bone-level comparisons.

## Conclusion:

latform switching consistently resulted in reduced marginal bone loss compared with platform-matched abutments throughout the 12-month follow-up period. The inward horizontal offset effectively preserved crestal bone by limiting microgap-related stress and inflammation. Therefore, platform-switched abutment designs can be considered a superior option for enhancing long-term implant stability and peri- implant tissue health.

## Figures and Tables

**Table 1:** Demographic characteristics of study participants

**Parameter**	**Platform-Switched (n = 50)**	**Platform-Matched (n = 50)**	**p-value**
Mean Age (years)	42.6 ± 8.4	43.1 ± 9.1	0.78
Gender (M/F)	26 / 24	25 / 25	0.84
Implant Site (Maxilla/Mandible)	28 / 22	27 / 23	0.9

**Table 2:** Clinical parameters during follow-up

**Parameter**	**Group**	**Baseline**	**3 Months**	**6 Months**	**12 Months**	**p-value**
Plaque Index	PS	0.42 ± 0.08	0.45 ± 0.10	0.47 ± 0.11	0.48 ± 0.09	0.12
	PM	0.40 ± 0.10	0.44 ± 0.09	0.46 ± 0.12	0.49 ± 0.10	0.15
Bleeding on Probing (%)	PS	6.2 ± 1.1	6.8 ± 1.3	7.1 ± 1.5	7.4 ± 1.3	0.09
	PM	6.5 ± 1.3	7.0 ± 1.4	7.6 ± 1.6	7.9 ± 1.4	0.11
Probing Depth (mm)	PS	2.12 ± 0.26	2.18 ± 0.24	2.22 ± 0.28	2.25 ± 0.30	0.2
	PM	2.10 ± 0.30	2.20 ± 0.27	2.29 ± 0.29	2.32 ± 0.30	0.18

**Table 3:** Comparison of marginal bone loss between groups

**Time Interval**	**Platform-Switched (mm)**	**Platform-Matched (mm)**	**p-value**
Baseline	0	0	-
3 Months	0.21 ± 0.08	0.38 ± 0.12	<0.001*
6 Months	0.36 ± 0.11	0.63 ± 0.18	<0.001*
12 Months	0.52 ± 0.14	0.89 ± 0.21	<0.001*
*Significant at p < 0.05

**Table 4:** Intragroup comparison of bone loss over time

**Group**	**Baseline → 3 Months**	**3 → 6 Months**	**6 → 12 Months**	**Overall p-value**
Platform-Switched	+0.21 mm	+0.15 mm	+0.16 mm	<0.001*
Platform-Matched	+0.38 mm	+0.25 mm	+0.26 mm	<0.001*
